# Round the Hospitals

**Published:** 1918-02-09

**Authors:** 


### ROUND THE HOSPITALS.

The article on "The Probationer's Third Year," which we published on page 379 of our issue last week, has apparently caused some misapprehension. We have always maintained and held that the training of nurses is an art which requires that each probationer shall have adequate time and continuous instruction, under the same conditions, in the wards, in the theatre, and in other departments. In too many hospitals the practice pursued has, in fact, subordinated the instruction to the circum-

☛ Previous articles appeared January 19 and 26.

**ROUND THE HOSPITALS—(*continued*).**

stances of the hospital, and the pressure of work throughout the hospital, whereby a probationer may be moved frequently from one ward to another, and so training of the best, and indeed, in our view, training at all that is worthy of the name, may for practical purposes become a secondary consideration, if not an impossibility. For instance, every competent sister has her own plan of instructing the nurses in her ward, and no instructor, however able, can possibly give a probationer adequate instruction, when she only has her in the ward for periods of a week, or for any period, in our view, which does not extend to two months at the least. Three months, in our view, is the ideal for every nurse-training school to aim at securing, and in no case should a sister have less than two months during which she has the care and instruction of each probationer sent to her ward.

It may be unique, for example, for a hospital to move its probationers frequently from ward to ward. We wish we could believe it was unique, because then there would only be one, instead of, as we fear there may be, a number of hospitals at present, which permit the claims of the probationers to adequate instruction, to become subordinate to the work which has to be provided for in the particular hospital. In the same way, and we want to be quite clear on this point, we are convinced that the nurse-training schools of the best are absolutely right in maintaining that two years' instruction under any circumstances is insufficient, and that in the third year, or third and fourth years, the probationers ought not to be utilised in the service of the hospital to earn fees by being occupied, in behalf of the hospital, on the private nursing staff. We have no desire to reopen a controversy which has been threshed out to a conclusion. Indeed, these points, so far as British nursing is concerned, have undoubtedly been settled. Our present desire is to make it perfectly clear where we stand, in regard to the responsibility upon each hospital which takes probationers, to provide that they shall be thoroughly trained. Further, that during their time of training each probationer shall be safeguarded from being overworked, because the hospital happens to be full, and so adequate training, in the highest and best meaning of the word, becomes a secondary matter. That is, in plain English, as far as the probationers' interests are concerned, they are not safeguarded from defects due to incompleteness of system and prohibitive conditions. The war has greatly increased the injustice under which large numbers of probationers, in some hospitals, are labouring. It is the plain duty of the chairman of every committee to make himself acquainted with the actual condition of affairs in any hospital for which he may be responsible. It is his privilege to satisfy himself, that the work of training probationers is not neglected, though it is war-time, and that the reputation of his hospital, and the future of all probationers working within its walls, are not imperilled by the absence of a system of adequate training at the present time, including the necessary theoretical instruction by the Medical Staff and the regular teachers.

We welcome authoritative and demonstrable evidence that the self-denying work of the British Women's Hospital Committee, under the leadership of Lady Cowdray, who, with her associates, is rendering splendid personal service in the matter, has awakened the deepest interest and appreciation amongst the great body of nurses everywhere. We are glad to be in a position to record that an ever-increasing number of trained nurses have written to applaud and support the objects of the College of Nursing, and to testify to the wisdom of the British Women's Hospital Committee in raising funds for its endowment and buildings. Not only have members of the nursing profession in considerable numbers spontaneously afforded this testimony, but very many trained nurses are continuously sending subscriptions to the Fund, so that all grades of nurses are now joining hands with the British Women's Hospital Committee. This spontaneous testimony has cheered Lady Cowdray and her fellow-workers, as well it may do, seeing that the funds they have started are especially organised to enable laymen to have the privilege of helping the nurses to help themselves by speedily being provided with a centre in the form of adequate College buildings, through which the education, training, and progressive development of nursing in every direction may extend and flourish.

The war has called forth the admiration of people of all classes throughout the Empire for our nurses and their services, whether given in hospitals abroad or adjacent to the fighting-line, in Territorial and other military hospitals, or in the voluntary hospitals at home. So excellent has the service been that everywhere people gladly confess to a desire to do anything in their power to advance the well-being and independence of the trained nurse, to help her on her way, and to provide as far as possible for the placing at her disposal of every educational and other technical advantage calculated to improve her future outlook, and to equip her to the fullest extent for the adequate discharge of the responsible duties to the sick which it is her privilege to render. Another interesting fact in this connection is that the British Women's Hospital Committee's report that certain journals of nursing have already sent handsome donations, while private nurses, probationers in training-schools, nursing staffs, medical staffs, Boards of Guardians, and other officials are sending in subscriptions continuously. This representative participation in gifts to the Fund now being raised for the College assures the Committee that its present purpose has behind it the convinced support of the great majority of the members of the nursing profession.

We are glad to note that the number of letters written by patients in testimony to the devoted and invaluable service rendered by trained nurses to wounded warriors is growing continuously. A

**ROUND THE HOSPITALS—(*continued*).**

subaltern, in the course of his communication, declares: "The nurses are simply splendid; I shall never forget how good they have been to me." A captain who has been suffering from his wounds for eighteen months, and so can appreciate the work done by his excellent nurses, testifies that he "is one of those who have known what a trained nurse's care and everlasting watchfulness mean" to the suffering warrior. A captious critic has complained of the advertisements which have appeared of the Nation's Fund for Nurses, being a Tribute from the British Empire to British Nurses. Such criticism is notoriously green in colour, and, if any justification for the advertisements is needed, we have it to-day in the form of a letter asking, on behalf of an officer, to whom he can send a contribution to this Fund, as there are so many officers who will be glad to give, if we can inform them where to send their gifts. The newspapers are difficult reading for busy people nowadays, so we may add the information that all contributions should be sent to the British Women's Hospital Committee, 21 Old Bond Street, London, W. 1. Further, our correspondent may be glad to hear that another suggestion—namely, the publication in the *Times* of the above information—has already been carried out several times during the last six weeks. We hope that many people of both sexes, who take an interest in our voluntary and military hospitals in the Homeland, will exercise their privilege by promptly sending a personal gift to the Nation's Fund for Nurses.

OUR trained nurses have shown great heroism often, the latest occasion being that of the sinking of two British ships in the Mediterranean, when eight nurses on one of the ships lost their lives. The names of these nurses appeared in the casualty lists on February 5, and their nearest relatives will receive in due course the commemorative medal to be issued in respect of all who have fallen in the war. Our women have taken a noble part in many fields connected with the present war, and it seems to us that if there can be comparison in the strength of feeling in regard to individual conduct by women workers, then there is not a single hospital supporter—indeed, we may add a single patient—who has had the privilege of being associated with a voluntary or other hospital within the Empire who will not claim the privilege of sending quickly their personal gift to the Nation's Fund for Nurses, and so ensure that their Tribute as a member of the British Empire shall help and encourage British Nurses.

SOME nurses have broken out into verse since the war, and have even printed their lines. It may be well to point out, therefore, that no steps to sell products of this kind, or to raise money by this or other means for the Nation's Fund for Nurses, can properly be taken until Mrs. Smeaton Douglas, the secretary of the British Women's Hospital Committee, 21 Old Bond Street, London, W. 1, has been apprised of the wish to help and the approval of the Committee has been obtained.

A GOOD many hospital people may like to be reminded of the meeting in aid of the British Women's Hospital appeal for the Nation's Fund, to be held on February 8, at 2.30 P.M., at the West End Cinema, Coventry Street, W., when the official Red Cross film will be presented for the first time, in conjunction with the great new Admiralty picture "Our Naval Air Power." This is the first opportunity the public has had to see this Red Cross film. It is a fine picture of about 2,000 feet in length, and exhibits the progress of the wounded warrior from the battlefield to a hospital in the Homeland. It commences with the regimental stretcher-bearers going into No Man's Land to bring in the wounded patient, the latter's subsequent journey to the regimental aid-post, then from the trenches to the dressing station, thence by motor ambulance to the Red Cross train, and so on to the port where he is transferred to the hospital ship, is landed in England, his journey in the hospital train, and his arrival at the Home hospital. Friday's gathering in Coventry Street will hear some interesting facts from the Duchess of Bedford and others, including Mr. Pett Ridge and Mr. Ben Tillett, M.P., in relation to the work of our nurses. Miss Lilian Braithwaite will recite the "Mercy Speech" from the "Merchant of Venice," and will be followed by Madame Blanche Marchesi and Mr. Thorpe Bates, who will sing. On Saturday Madame Kirkby Lunn will hold a recital at the Æolian Hall, New Bond Street. There will be sixteenth- and seventeenth-century English songs, old Breton folk-songs, and some modern English and French songs. Madame Lunn's vocal recital also is in aid of the Nation's Fund for Nurses, which includes an endowment for the College of Nursing, with scholarships to encourage study in the higher branches of the profession, and a Tribute Fund for nurses in sickness and old age.

OUR readers have become accustomed to, and even possibly weary of, the reiterated misstatements on the part of opponents of the College. It may interest them, however, to point out that if, as these opponents maintain, the presence of men upon the governing body of a profession, who are not members of that profession is an indication of lay control, and if doctors are to be regarded as employers of nurses, then the General Council provided for in the Bill for the State Registration of Nurses, drawn up by the Central Committee for State Registration, will most certainly be under lay control, and under the control of employers too. The Bill provides for the formation of a Council of thirty-three persons, of whom three are to be appointed by the Privy Council, three to be registered medical practitioners appointed by the Local Government Boards of England, Scotland, and Ireland respectively, three to be registered practitioners appointed by the British Medical Association, one a registered practitioner appointed by the Medico-Psychological Association, and one a registered practitioner appointed by the medical superintendents of fever hospitals. Now the Privy Council can in its wisdom, if it so decides, appoint

**ROUND THE HOSPITALS—(*continued*).**

three laymen, and there will also be eight doctors who are employers of nurses. As evidence of the extraordinary blindness to the truth and true inwardness of a position, it may interest our readers to give the following quotation from a letter written by the honorary secretary of one of the societies affiliated to the Central Committee. Here it is: "Anyone who has worked on a committee with both doctors and nurses knows that their weight as employers is overpowering, and that it takes a very strong-minded rank-and-filer to oppose them. One does not blame either side for this, it is just human nature; but the organisation of the College is branded as undemocratic in consequence"! No stronger evidence could be afforded of the notorious fact that the opposition to the College does not rest upon reason or fact, but is the work of a prejudiced few who are obsessed by an idea, and have axes of their own to grind, which are not calculated to further the best interests and progress of the nursing profession.

---

The war has taught even the most experienced and successful raiser of funds for hospitals and approved charities much that would have seemed incredible before the war. Organisers of work guilds, the almoners' work at our hospitals, and a multiplicity of other things which may add to the efficiency and extend the area of work of the voluntary hospitals and their beneficial influence, might learn a great deal in the way of raising funds, if they would keep their eye upon parish magazines, and all sorts of out-of-the-way channels of information which record what is being done by small communities up and down the country. In Northamptonshire the small village of Sudborough, situated in a rural district miles away from any station, which contains some 150 inhabitants all told, was fired by an enterprising spirit with the idea of accepting an invitation issued by Lord Spencer in support of his scheme to raise £15,000 from the town and county of Northampton for the Red Cross. Steps were at once taken to organise the village, and within a week the appointed day for a Red Cross garden party in the Vicarage garden arrived. Everybody, high and low, rich and poor, young and old, resolved to be present and contribute, directly and indirectly, all they could to make that garden party memorable. August 23, the appointed day, proved uninviting, with frequent downpours of rain, but the spirit and will to embrace this opportunity of helping a cause dear to the hearts of all was so strong, that there was not a man, woman, or child in the village who did not make it their business to be present and to give according to their means.

---

The more desperate grew the weather, the greater was the energy of the benevolent spirit which possessed those who took part in "the Day" at Sudborough. There was a whist drive, which yielded £2 14s. 9d., a pound stall which yielded £3 10s. 3d., a post-office 14s. 8d., competitions £15 5s. 6½d., raffles £5 3s. 7d., an auction £19 5s. 11d., paper (sold) £2 12s. 1d., gate and bicycles £2 0s. 9½d., refreshments £6 3s. 11d., and voluntary contributions amounting to £41 18s. 6d., making a total with sundries of no less than £100. We record this village fête, and the facts that refreshments and labour, including the putting up of games and competitions, the typing and writing out of notices, and all expenses of every kind connected with it were all given, with supreme pleasure. Townspeople are apt to smile at the want of energy of village communities. Is not this instance evidence that the boot is on the other leg? Further that what the villages have lacked in the past has been the opportunity, and not the will, to render personal service, and do good deeds with splendid energy and completeness. Let us remember for the future that England and Wales contain some fourteen thousand villages, of which Sudborough is but one, and possibly amongst the smallest, of this number. Bedfordshire, as we testified some time ago, has discovered a mine of value in each English village, and Cambridgeshire soon followed suit. Will the year bring into bearing the villages which remain? Example, we confess, is better than precept, but we decline to apologise in the circumstances.

---

Owing to the dearth of nurses, V.A.D.s have for some time been assisting at Queen Mary's Hospital, one of the institutions of the Metropolitan Asylums Board. Last June the Board agreed to make a contribution of five guineas a month to the funds of the Surrey branch so long as it continued to send about eight V.A.D. nurses daily to assist. The medical superintendent reports that the aid of the V.A.D.s is much appreciated, and the Asylums Board has decided to continue the arrangement. It is just to state that the five guineas a month merely covers travelling and other out-of-pocket expenses.

---

The governors of the Hartlepools Hospital have decided to give a war bonus of £5 to each of the nurses who has been in the employment of the managers for one year. It is not apparent whether the hospital has found it difficult to maintain its nursing staff and to keep free from a succession of changes. This has been the case in some institutions, but in any event the decision will be appreciated, whatever the reason which gave rise to it.

---

**It is our invariable practice to pay for all items of news which we may publish. The minimum payment is 5s. Paragraphs of about twenty lines in length—that is to say, of 150 words or less—are preferred. Contributions should be addressed to The Editor, The Hospital, 28 and 29 Southampton Street, Strand, London, W.C. 2, and, to be in time for the current week's issue, should reach him by the first post on Mondays.**

